# Immune signaling pathways in *Rhodnius prolixus* in the context of *Trypanosoma rangeli* infection: cellular and humoral immune responses and microbiota modulation

**DOI:** 10.3389/fphys.2024.1435447

**Published:** 2024-08-15

**Authors:** Suelen Bastos Pereira, Débora Passos de Mattos, Marcelo Salabert Gonzalez, Cicero Brasileiro Mello, Patrícia Azambuja, Daniele Pereira de Castro, Cecília Stahl Vieira

**Affiliations:** ^1^ Laboratório de Bioquímica e Fisiologia de Insetos, Instituto Oswaldo Cruz (IOC/Fiocruz), Rio de Janeiro, Brazil; ^2^ Programa de Pós-Graduação em Ciências e Biotecnologia, Universidade Federal Fluminense, Niterói, Brazil; ^3^ Universidade Federal Fluminense, Instituto de Biologia, Departamento de Biologia Geral, Laboratório de Biologia de Insetos, Niterói, Brazil; ^4^ Instituto Nacional de Entomologia Molecular (INCT-EM), Rio de Janeiro, Brazil; ^5^ Department of Parasitology, Faculty of Science, Charles University, Praha, Czechia

**Keywords:** *Trypanosoma rangeli*, *Rhodnius prolixus*, microbiota, immunity, signaling pathways

## Abstract

**Introduction:**

*Rhodnius prolixus* is a hematophagous insect and one of the main vectors for *Trypanosoma cruzi* and *Trypanosoma rangeli* parasites in Latin America. Gut microbiota and insect immune responses affect *T. cruzi* and *T. rangeli* infection within triatomines. Particularly the Toll and IMD signaling pathways activations and how they orchestrate the antimicrobial peptides (AMPs) expressions in *R. prolixus*, especially when infected by *T. rangeli*.

**Objectives:**

Examine how *T. rangeli* infection modulates *R. prolixus* cellular and humoral immunity and its impacts on insect microbiota.

**Methods:**

*R. prolixus* was fed on blood containing epimastigotes of *T. rangeli*, and infection was quantified in insect tissues. The gene expression of *dorsal*, *cactus*, *relish*, *PGRP*, and AMPs was examined in the midgut, fat body, and salivary glands by quantitative real-time PCR. Microbiota composition was analyzed using RT-q PCR targeting specific bacterial species. Hemocyte numbers and phenoloxidase activity were quantified to assess cellular immune responses.

**Results:**

*T. rangeli* infection modulated triatomine immunity in midgut and hemocoel, activating the expression of the NF-kB gene *dorsal*, associated with the Toll pathway; increasing expression of the gene encoding *PGRP* receptor, a component involved in the IMD pathway, both in the intestine and fat body; repressing the expression of the *relish* transcription factor, mainly in salivary glands. Among the *R. prolixus* AMPs studied, *T. rangeli* infection repressed all AMP gene expression, other than *defensin C* which increased mRNA levels. The PO activity was enhanced in the hemolymph of infected insects. *T. rangeli* infection did not induce hemocyte number alterations compared to control insects. However, an increase in hemocyte microaggregation was detected in infected insects.

**Discussion:**

*R. prolixus* recognizes *T. rangeli* infection and triggers humoral and cellular immune responses involving Toll pathway activation, *defensin C* synthesis, increased phenoloxidase activity, and enhanced hemocyte aggregation. On the other hand, *T. rangeli* infection suppressed some IMD pathway components, suggesting that, in *R. prolixus*, this pathway is involved in *defensins A* and *B* gene regulation. Importantly, these immune responses altered the bacterial microbiota composition, potentially favoring *T. rangeli* establishment in the insect vector.

## Introduction

The hematophagous insect *Rhodnius prolixus* is an important triatomine vector for *T. cruzi*, Latin America’s causative agent of Chagas disease. This species has the propensity to inhabit synanthropic environments, presents a fast life cycle, high population density and is very susceptible to *Trypanosoma cruzi* infection (Guhl et al., 2007; Coura and Junqueira, 2015). The genus *Rhodnius* is also known to be the natural invertebrate host for another Trypanosomatidae, the *T. rangeli* (Eger-Mangrich et al., 2001; Guhl and Vallejo, 2003; Urrea et al., 2005). *T. rangeli* displays varying degrees of pathogenicity in its vector, inducing behavioral and physiological variations (Andrade et al., 2023; Duarte da Silva and Guarneri, 2023).

Triatomines and mammals, including chronic Chagas disease patients, can be naturally coinfected by *T. rangeli* and *T. cruzi* (de Sousa et al., 2008). When identifying vector infections, the co-infection may promote difficulty in distinguishing parasite strains and, therefore, lead to incorrect identification of the etiological agent of Chagas disease (Ramirez et al., 2002; Castillo-Castañeda et al., 2022; Herrera et al., 2022; Vergara-Meza et al., 2022).

*T. cruzi* and *T. rangeli* present different life cycles in the vector, where the latter develops into metacyclic trypomastigotes in insect salivary glands. These infective forms are inoculated in vertebrate hosts through insect bites (Eger-Mangrich et al., 2001; Guhl and Vallejo, 2003). *T. rangeli* life cycle within the invertebrate host begins on trypomastigotes ingestion from infected vertebrates’ bloodstream. Following a brief interval post-infection, within the insect midgut, parasites undergo differentiation into epimastigotes, in its replicative form. These epimastigotes could cross the intestinal epithelium *via* an intracellular pathway, subsequently accessing the hemocoel within a timeframe ranging from 24 h to several weeks, depending on the parasite strain (Vallejo et al., 2009). In the hemolymph, *T. rangeli* continues multiplying freely or inside hemocytes, being able to invade salivary glands later on, where it multiplies and transforms into metacyclic trypomastigotes (de Oliveira and de Souza, 2001; Guarneri and Lorenzo, 2017) (Supplementary Material 2).

*T. rangeli* must overcome the immune responses of midgut, hemocoel, and salivary glands to complete its life cycle in the invertebrate host (Garcia et al., 2009). In the midgut, the parasite faces digestive factors, intestinal microbiota, and immune responses as obstacles. On the other hand, while in the hemocoel, *T. rangeli* must survive not only the humoral but also the cellular immune responses promoted by hemocytes (Mello et al., 1995; Azambuja et al., 1999; Garcia et al., 2004).

During an infection process, the invading microorganism is recognized by pathogen recognition patterns, which bind to pathogen-associated molecular patterns (Gillespie et al., 1997; Gottar et al., 2006). This process triggers the activation of different immune signaling pathways that increase the vector’s defense responses, both cellular and humoral (Salcedo-Porras and Lowenberger, 2019). In *R. prolixus*, the main signaling pathways are Toll, which involves the Dorsal transcription factor (TF) and its inhibitor cactus, and the immunodeficiency pathway (IMD), including the Relish TF (Stöven et al., 2000; Zasloff, 2002; Vieira et al., 2018). Essential pathogen recognition patterns related to the IMD pathway are peptidoglycan recognition proteins (PGRPs) that recognize Gram - bacteria (Ferrandon et al., 2007). These pathways, when activated, lead to antimicrobial peptide (AMP) expressions (Bulet et al., 1999; Vieira et al., 2015) such as defensins (A, B, and C) (Lambert et al., 1989; Dimarcq et al., 1990; Vieira et al., 2015; 2016) and prolixicin (Ursic-Bedoya et al., 2011).

*R. prolixus* has a rich microbiota (Azambuja et al., 2005), composed of a wide variety of microorganisms, including commensal bacteria, such as *Rhodococcus rhodnii*, a symbiont that plays an essential role in vector physiology, helping in nutrition and vitamin production from complex B (Dillon and Dillon, 2004; Batista et al., 2021). Some strains of *Serratia marcescens,* a gut commensal bacteria of *R. prolixus* (Azambuja et al., 2004; da Mota et al., 2012; Da Mota et al., 2019), produce a hemolytic factor aiding blood digestion and exhibiting effective lytic activity against trypanosomatids (Castro et al., 2007a; Castro et al., 2007b; Da Mota et al., 2019). According to our previous work, *T. rangeli* Macias infection activates AMP gene expression in the insect midgut, impacting gut microbiota by reducing Enterococcaceae levels (Vieira et al., 2015). Hence, digestive tract commensal microbiota can be affected by vector immune system activation post-parasite exposure, therefore influencing the success of the parasite infection (Castro et al., 2012; Vieira et al., 2015; 2016; Batista et al., 2021).

Regarding the tripartite interaction, parasite-insect-microbiota, to complete its biological cycle, *T. rangeli* needs to overcome the vector humoral and cellular immunity in addition to dealing with the intestinal microbiota, including *S. marcescens* that could promote epimastigote lysis. Thus, understanding the modulation of insect immune responses during tripartite interaction will favor identifying new parasite-killing potential targets.

## Materials and methods

### *Rhodnius prolixus* colony maintenance and ethics statement

*R. prolixus* was reared at Laboratório de Bioquímica e Fisiologia de Insetos, Instituto Oswaldo Cruz (IOC/Fiocruz) under a relative humidity of 50%–60% at 27°C (Azambuja and Garcia, 1997). Four weeks starved fifth instar nymphs were randomly chosen and fed in an artificial feeding system with defibrinated rabbit blood (Azambuja and Garcia, 1997). The rabbit blood taken by cardiac puncture was provided by the Instituto de Ciência e Tecnologia em Biomodelos, following the Ethical Principles in Animal Experimentation and approved by the Comissão de Ética no Uso de Animais (CEUA/Fiocruz, under the protocol number LW019/17).

### *Trypanosoma rangeli* culture

*T. rangeli* Macias strain epimastigotes were cultivated in Brain Heart Infusion media (Sigma-Aldrich, São Paulo, Brazil) supplemented with 20% heat-inactivated sterile-filtered bovine fetal serum at 28°C, under sub-cultivation twice a week (Vieira et al., 2015). As documented previously, *T. rangeli* Macias was selected based on its infectivity rate and capacity to invade *R. prolixus* hemocele (Machado et al., 2006).

### *R. prolixus* infection with *Trypanosoma rangeli*

*R. prolixus* fifth instar nymphs were randomly selected and were infected with *T. rangeli.* Epimastigotes in the exponential growth phase were quantified in a Neubauer chamber under a phase contrast optical microscope. Defibrinated rabbit blood was previously centrifuged at 2,000 x g for 15 min at 4°C, and the supernatant (plasma) was collected and incubated at 55°C for 30 min to inactivate the plasma complement system (Vieira et al., 2015). Afterward, the plasma was mixed with the blood erythrocytes after adding epimastigotes in a final concentration adjusted to 1 × 10^6^ epimastigotes/mL blood. The blood offered to control insects received the same volume of Brain Heart Infusion medium culture instead of epimastigotes. The blood containing or not *T. rangeli* was offered to *R. prolixus* through the artificial membrane-feeding apparatus at 37°C.

### *Trypanosoma rangeli* detection and quantification in *Rhodnius prolixus* tissues

In three experiments, 52 insects infected with *T. rangeli* were dissected 7, 15, 21, and 29 days after feeding/infection (DAF). Insect’s hemolymph, midgut, and salivary glands were individually collected, placed in 1.5 mL microtubes, and homogenized in PBS to confirm and quantify *T. rangeli* infection. Parasites were counted using a Neubauer chamber under a light contrast microscope and expressed as parasites/mL. For parasite quantification in hemolymph samples, the insects had their first pair of legs cut off, and individual drops of hemolymph were immediately stored in 1.5 mL microtubes. For parasite detection in *R. prolixus* salivary glands, the glands were dissected by removing the pronotum using tweezers and scissors to expose the pair of salivary glands. The tissue was stored in 1.5 mL microtubes containing 50 µL PBS and macerated with pistils. To collect the midgut, the abdominal cuticle was removed, and midgut samples were collected separately, each placed in sterile 1.5 mL microtubes containing 90 µL PBS.

### *R. prolixus* tissue collection for microbiota and molecular biology analysis

Midgut, fat body, and salivary glands were collected 1 and 7 days after *T. rangeli* infection in three pools of five insects (*n* = 15), as described above. However, *R. prolixus* salivary glands were only collected 7 days after feeding with *T. rangeli*, considering the time the parasite takes to invade the insect hemolymph as well as salivary glands, as described previously (Hecker et al., 1990; Garcia et al., 2012; Paim et al., 2013; Ferreira et al., 2015).

The collection of *R. prolixus* tissues for qPCR and microbiota analysis was carried out under sterile conditions. Microbiota was studied in midgut samples 7 DAF. Tissues were immediately placed in sterile, empty 1.5 mL tubes and then conditioned in dry ice, for rapid freezing and stored at −80°C.

### Gene expression quantification

Gene expression analyses were carried out using cDNA from the fat body, midgut, and salivary glands of *R. prolixus*. Firstly, total RNA was extracted and quantified using the NucleoSpin ^®^ RNA II kit (Macherey-Nagel) and the NanoDrop 2000 (Thermo Scientific) respectively. The cDNA was made using the First-Strand cDNA Synthesis kit (GE Healthcare), following the manufacturer’s protocol, from 2.5 µg total RNA. For RT-qPCR, the GoTaq^®^ qPCR Master Mix kit (PROMEGA) used to analyze the expression of AMPs (*RpdefA, RpdefB, RpdefC, Rpprol*) and genes related to the Toll and IMD pathways (*Rpdorsal, Rpcactus,* and *Rprelish*), which were normalized based on constitutive *R. prolixus* genes expression (*α-tubulin* and *GAPDH*). The specific primers for the genes of interest, as well as the constitutive genes of *R. prolixus*, were designed and used as previously published: *α-tubulin* and *GAPDH* (Paim et al., 2012), *RpdefA*, *RpdefB* and *RpdefC* (Lopez et al., 2003; Vieira et al., 2016), *Rpprol* (Ursic-Bedoya et al., 2011; Vieira et al., 2016), *Rpcactus* (Ribeiro et al., 2014), *Rprelish, Rpdorsal* (Mesquita et al., 2015).

For microbiota analysis, the relative expression of *S. marcescens*, *R. rhodnii*, and Enterococcaceaee 16S-rRNA genes was analyzed in midgut samples collected at 7 DAF as previously described (Vieira et al., 2016). These bacteria species were chosen based on relevant evidence from previous publications (Vieira et al., 2015; da Mota et al., 2019). Bacterial 16S-rRNA and *R. prolixus* gene expressions were calculated using the ΔΔCT (relative quantification) method (Livak and Schmittgen, 2001). The RT-qPCR reaction was performed on the 7,500 equipment (Applied Biosystems). PCR details: initial denaturation at 95°C for 20 s, denaturation at 95°C for 3 s, annealing and extension for 30 s, repeated 40 times. The final extension occurred at 72°C following a melting curve analysis. Secondary analyses were carried out based on Vieira (2016), using the Expression Suite v1.0.3 software (Life Technologies), considering the amplification efficiency of each target (Vieira et al., 2016; 2018). Primers used in the present work are described in Supplementary Table.

### Hemocyte and nodule quantification

*R. prolixus* hemolymph samples (10 µL) were individually collected, placed in 1.5 mL microtubes, and immediately mixed with 10 µL anticoagulant solution (0.01 M ethylenediamine tetraacetic acid, 0.1 M glucose, 0.062 M sodium chloride, 0.03 M trisodium citrate, 0.026 M citric acid, pH 4.6) (Garcia and Azambuja, 1991) at 2, 7, and 12 DAF. Hemocytes and nodule formations in 10 µL hemolymph/anticoagulant solution were quantified using a Neubauer chamber and a phase-contrast optical microscope. Only clusters from five or more hemocytes were considered as hemocyte microaggregation (Garcia et al., 2004).

### Phenoloxidase assays

Phenoloxidase (PO) activities were assessed in freshly collected hemolymph from the fifth instar nymphs at 7 and 12 DAF. 10 μL hemolymph volume was collected from individual insects and diluted in 200 µL ultrapure water, followed by centrifugation at 10,000 x g for 10 min. The supernatant was further diluted tenfold for analysis. Each group contained five insects, and the experiments were performed in triplicate (*n* = 15). Hemolymph samples (25 μL) were mixed with 10 μL cacodylate-CaCl_2_ buffer (10 mM sodium cacodylate, 10 mM CaCl_2_, pH 7.4) in 96-well plates. Subsequently, 25 μL saturated solution of DOPA (4 mg/mL) was added to each well and then incubated at 28°C in a Spectra Max 190 Microplate Reader (Molecular Devices, California, United States) at 37°C for 120 min. The absorbance at 490 nm, indicative of dopachrome formation from DOPA, was continuously monitored in the hemolymph samples. PO activity was quantified and expressed as absorbance/min × 100, then calculated based on the dopachrome formation, as formerly described (Genta et al., 2010).

### Trypanolytic activity

*S. marcescens* RPH1 was grown in Brain Heart Infusion Agar medium for 24 h. A colony was isolated and cultivated in 20 mL Brain Heart Infusion Broth for 18 h at 30°C and 90 rpm. The bacterial culture in the stationary growth phase was collected and adjusted to a concentration of 1 × 10^8^ CFU/mL. *T. rangeli* Macias epimastigotes were grown to a concentration of 2.5 × 10^6^ parasites/mL in the exponential growth phase and then incubated with *S. marcescens* RPH1 for 120 min at 30°C. After the incubation, motile parasites were counted using a Neubauer chamber and an optical microscope with phase contrast at ×40 magnification (Castro et al., 2007a).

### Statistical analysis

Results were analyzed by GraphPad Prism 5.0 program using 1 Way ANOVA, normality test, *t*-test (unpaired), or Mann Whitney test (nonparametric test) depending on data distribution and number of treatments. Differences between groups are considered not statistically significant when *p* > 0.05.

## Results

### *Trypanosoma rangeli* infection

*R. prolixus* infected with *T. rangeli* were dissected at 7, 15, 21, and 29 days after feeding/infection (DAF). Parasites were detectable in midgut samples starting at 15 DAF, with an average of 1,5 × 10^6^ parasites/mL in the digestive tract. At 29 DAF, we found 1 × 10^4^ parasites/mL in the salivary glands of two insects. Then, 55.8% of the nymphs were infected.

### Relative expression of humoral immunity genes in *R. prolixus* infected by *Trypanosoma rangeli*

**FIGURE 1:**
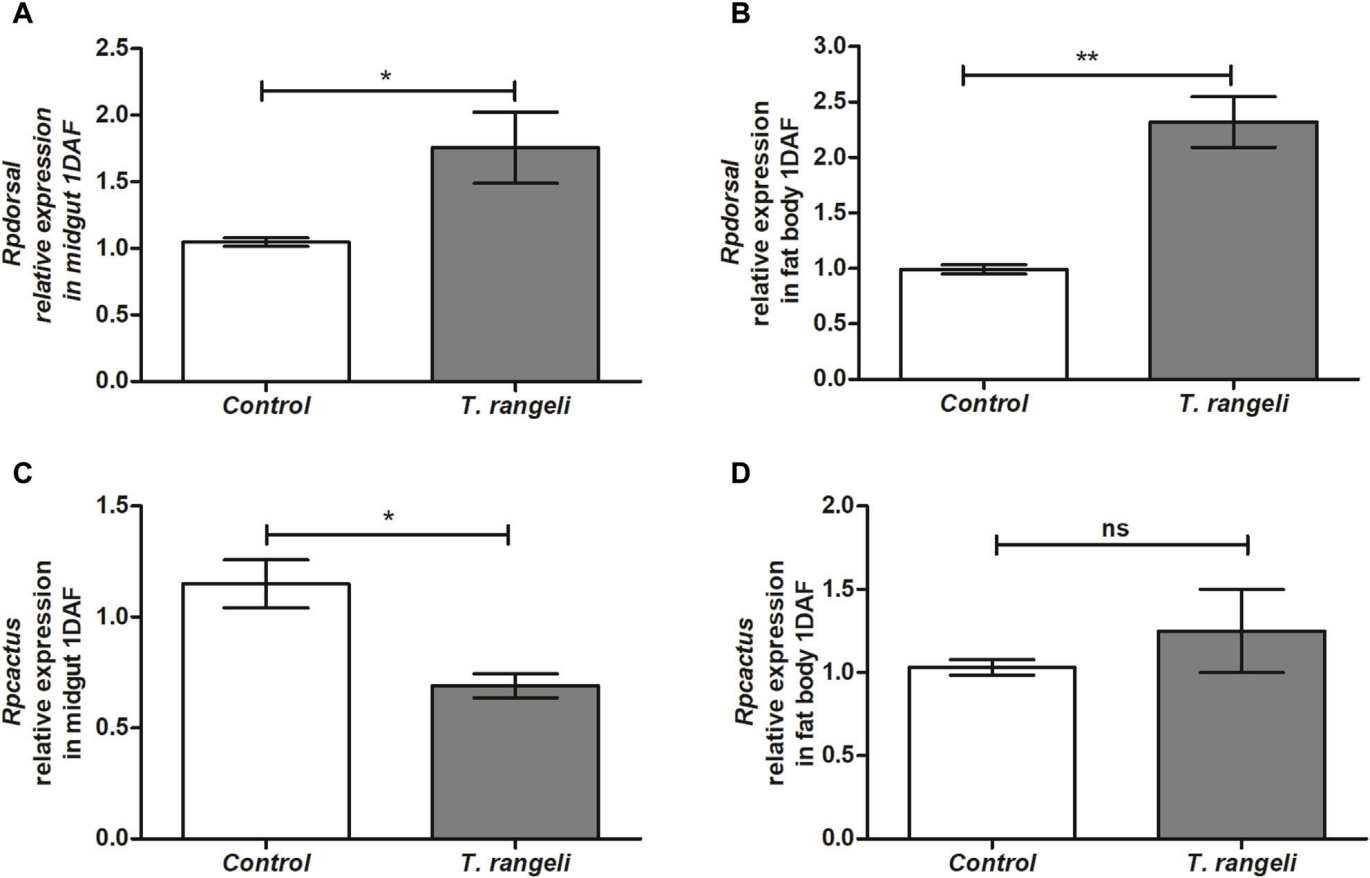
Relative gene expression of components from the Toll pathway in *Rhodnius prolixus* infected with *Trypanosoma rangeli*. *Rpdorsal* and *Rpcactus* gene expressions were analyzed using the midgut and fat body samples from *R. prolixus* fifth instar nymphs 1 day after feeding (DAF) with blood containing *T. rangeli* (10^6^ epimastigotes/mL). Data were quantified using the gene expression of uninfected insects fed on blood as the calibrator (white columns) in comparison with infected insects (gray columns) and shown as the relative expression of **(A)** *Rpdorsal* in anterior midgut, **(B)** *Rpdorsal* in the fat body, **(C)** *Rpcactus* in anterior midgut, **(D)** *Rpcactus* in the fat body. Bars represent the mean ± SEM of three independent experiments with three pools of insects (n = 3). Means were compared using Student’s T-test; *p < 0.05, **p < 0.01, ns = non-significant difference.

**FIGURE 2:**
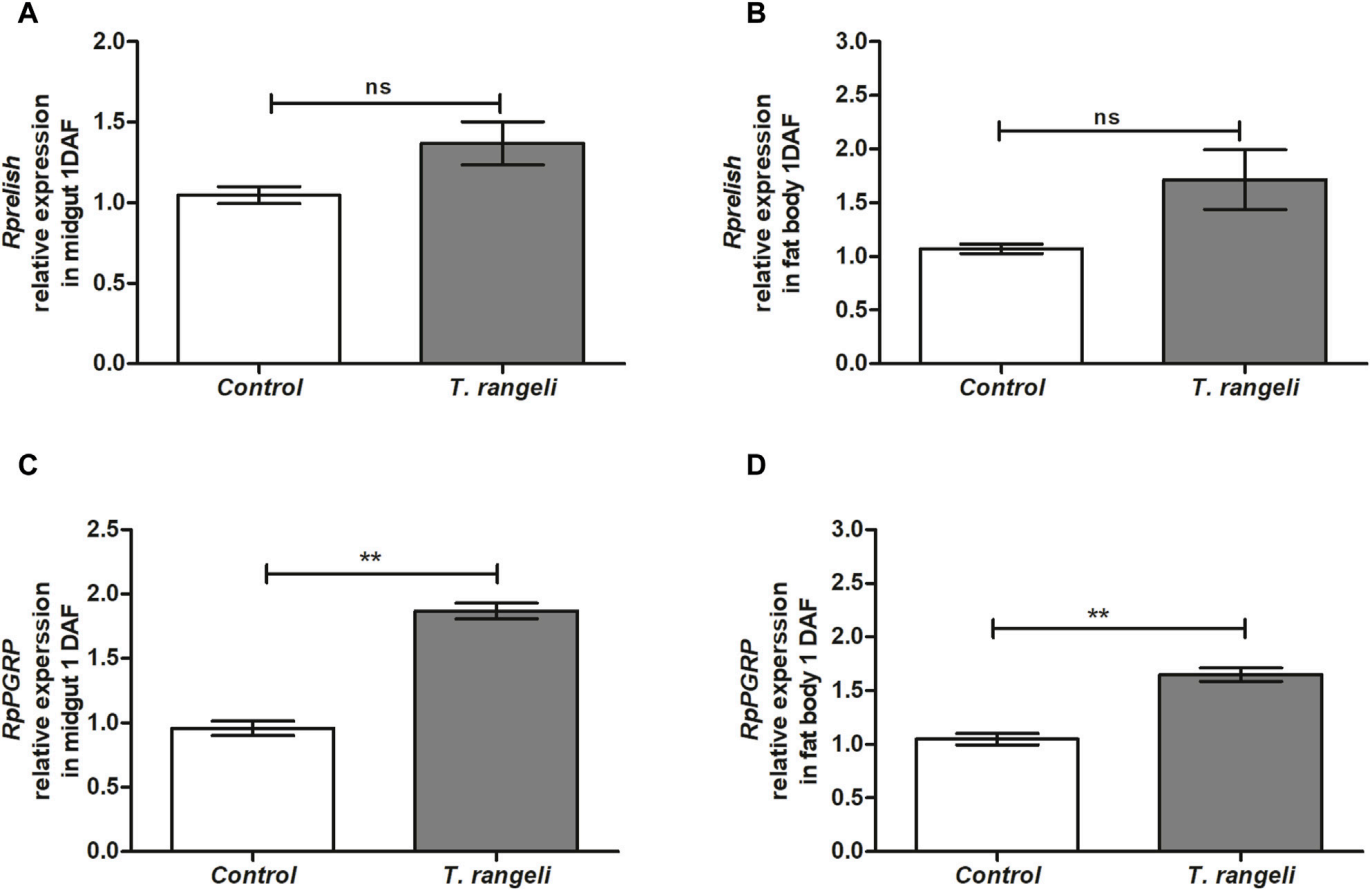
Relative gene expression of components from the IMD pathway in *Rhodnius prolixus* infected with *Trypanosoma rangeli*. *Rprelish* and *RpPGRP* gene expressions were analyzed using the midgut and fat body samples from *R. prolixus* fifth instar nymphs 1 day after feeding (DAF) with blood containing *T. rangeli* 106 epimastigotes/mL). Data were quantified using the gene expression of uninfected insects fed on blood as the calibrator (white columns) in comparison with infected insects (gray columns) and shown as the relative expression of **(A)** *Rprelish* in anterior midgut, **(B)** *Rprelish* in the fat body, **(C)** *RpPGRP* in anterior midgut, **(D)** *RpPGRP* in the fat body. Bars represent the mean ± SEM of three independent experiments with three pools of insects (n = 3). Means were compared using Student’s T-test; **p < 0.01, ns = non-significant difference.

*T. rangeli* infection modulated factors that regulate AMP transcription in different *R. prolixus* tissues. *Rpdorsal* TF expression, related to the Toll pathway, was significantly higher in the midgut (*p* < 0.05) and fat body (*p* < 0.01) of *T. rangeli*-infected insects than in control at 1 DAF, 2.5-fold higher in the latter (*p* < 0.01; Figure 1). On the other hand, *Rpcactus*, the Toll pathway inhibitor, was negatively modulated by *T. rangeli* infection in the insect midgut at 1 DAF (*p* < 0.05), but not in the fat body (Figure 1). The *Rprelish* gene expression, the IMD pathway transcription factor, was not significantly modulated by *T. rangeli* infection in both midgut or fat body at 1 DAF compared to non-infected insects (Figure 2). Although, we observed a high expression level of the *RpPGRP* gene receptor (a component of the IMD pathway) in the midgut (*p* < 0.01) and fat body (*p* < 0.01) on *T. rangeli*-infected insects 1 DAF (Figure 2).

### AMPs expression in the fat body of *R. prolixus* infected by *Trypanosoma rangeli*

**FIGURE 3:**
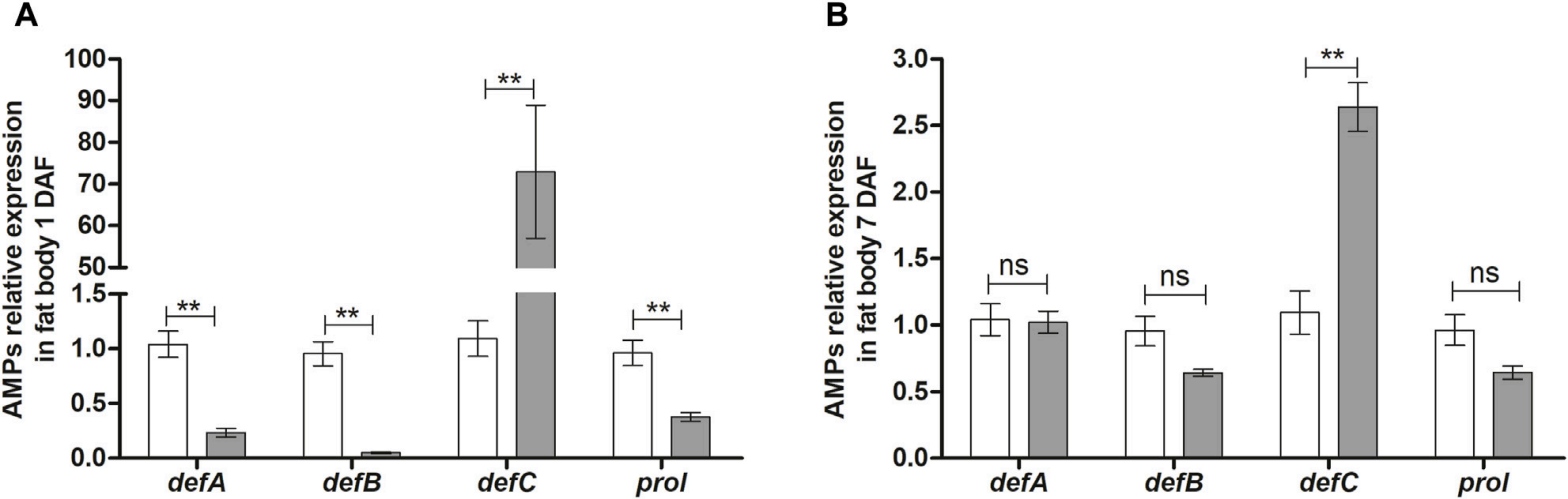
Antimicrobial peptides gene expression in the fat body of *Rhodnius prolixus* infected with *Trypanosoma rangeli*. *R. prolixus* fifth instar nymphs were fed on blood containing *T. rangeli* epimastigotes (10^6^ parasites/mL of blood). Data were quantified using the gene expression of untreated control insects as the calibrator (white columns). The gray columns show the relative expression of the antimicrobial peptide genes on the **(A)** 1st and **(B)** 7th days after feeding insects on blood containing *T. rangeli* epimastigotes. Relative gene expression of *RpdefA*, *RpdefB, RpdefC*, *Rpprol*. Each bar represents three independent experiments performed in duplicate (n = 6). Means were compared using one-way ANOVA and Student’s t-test; **p < 0.01, ns = non-significant difference.

In the fat body, the expression of the AMPs genes, *RpdefA* and *RpdefB,* as well as *Rpprol,* was reduced at 1 DAF (Figure 3A) but not changed at 7 DAF (Figures 3A, B). In contrast, AMP *RpdefC* expression in *T. rangeli* infected insects was 70 times higher (*p* < 0.01; Figure 3A) than control insects at 1 DAF and remained high (2.5-fold) at 7 DAF (*p* < 0.01 Figure 3B).

### Expression of NF-kB transcription factors and AMPs in the salivary glands of *R. prolixus* infected by *Trypanosoma rangeli*

**FIGURE 4:**
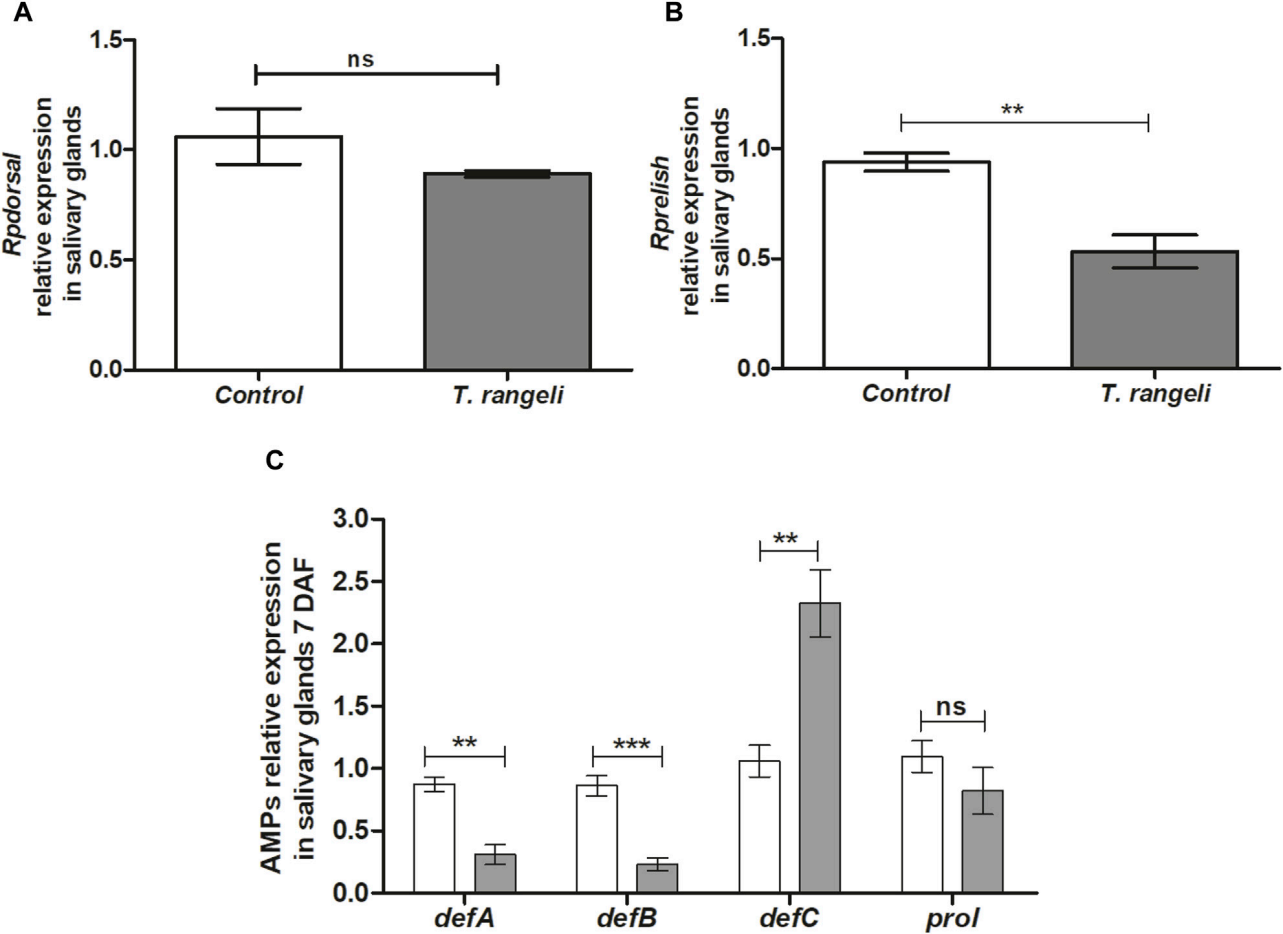
NF-kB transcription factors and antimicrobial peptides gene expression in the salivary glands of *Rhodnius prolixus* oral infected with *Trypanosoma rangeli*. Fifth instar nymphs of *R. prolixus* were fed on blood containing *T. rangeli* epimastigotes (10^6^ parasites/mL of blood). Data were quantified using the gene expression of untreated control insects as the calibrator (white columns). The grey columns show the relative expression of the **(A)** *Rpdorsal* **(B)**- *Rprelish* and **(C)** antimicrobial peptides genes (*RpdefA*, *RpdefB*, *RpdefC*, *Rpprol*), analyzed at 7 days after feeding (DAF). Each bar represents three independent experiments performed in duplicate (n = 6). Means were compared using one-way ANOVA and Student’s t-test; ***p < 0.001, **p < 0.01, ns = non-significant difference.

In *T. rangeli-infected* insect’s salivary glands, the TF Dorsal expression was not modulated at 7 DAF (Figure 4A). On the other hand, the *Rprelish* gene was downregulated in the same group (*p* < 0.01; Figure 4B). In the context of the AMP’s expression, *T. rangeli* infection induced suppression of *RpdefA* (*p* < 0.01) and *RpdefB* (*p* < 0.001) gene expression, while upregulated *RpdefC* (*p* < 0.01) expression in *R. prolixus* salivary glands at 7 DAF (Figure 4C). *Rpprol* was not modulated by parasite infection (Figure 4C).

### Modulation of hemocyte number and microaggregation in *R. prolixus* after *Trypanosoma rangeli* infection

**FIGURE 5:**
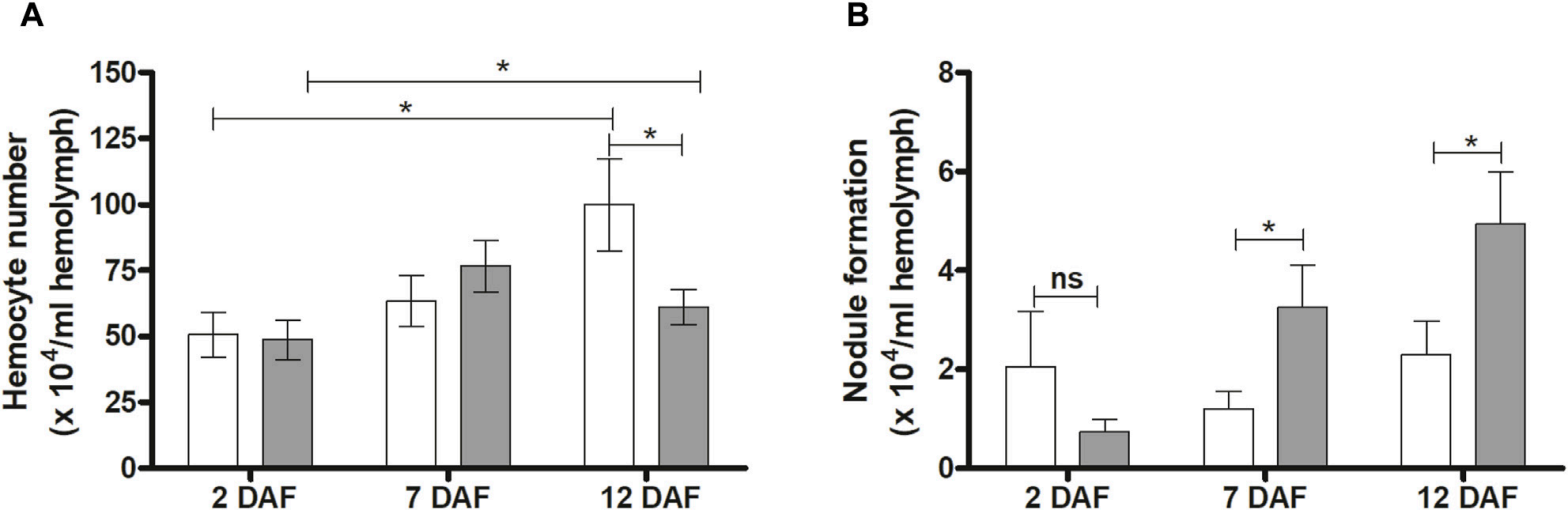
Effects of *Trypanosoma rangeli* infection on hemocytes and hemocyte microaggregation in *Rhodnius prolixus* hemolymph. The number of hemocytes **(A)** and hemocyte microaggregation **(B)** were counted in the hemolymph of *R. prolixus* fifth instar nymphs 2, 7, and 12 days after feeding (DAF) with blood containing *T. rangeli* epimastigotes (10^6^ parasites/mL). White column - control, non-infected insects; grey column - insects infected with *T. rangeli*. Each data represents the mean of three independent experiments with five insects in each group per day analyzed (n = 15). The vertical bars indicate the standard error (±EP). Controls and treatments were compared each day with a *t*-test or Mann-Whitney test. *p < 0.05 and ns = non-significant difference.

Hemocyte quantification and microaggregation in *R. prolixus* infected hemolymph were evaluated at 2, 7 and 12 DAF. No significant differences between control and infected insects were detected in hemocyte number at 2 and 7 DAF (Figure 5A). However, at 12 DAF, a significant reduction in this quantification was observed (*p* < 0.05) (Figure 5A). On the other hand, *T. rangeli* infection increased hemocyte microaggregation in *R. prolixus* hemolymph at 7 and 12 DAF (both *p* < 0.05) (Figure 5B), comparing with control uninfected insects.

### Phenoloxidase activity in the hemolymph of *R. prolixus* infected with *Trypanosoma rangeli*

**FIGURE 6:**
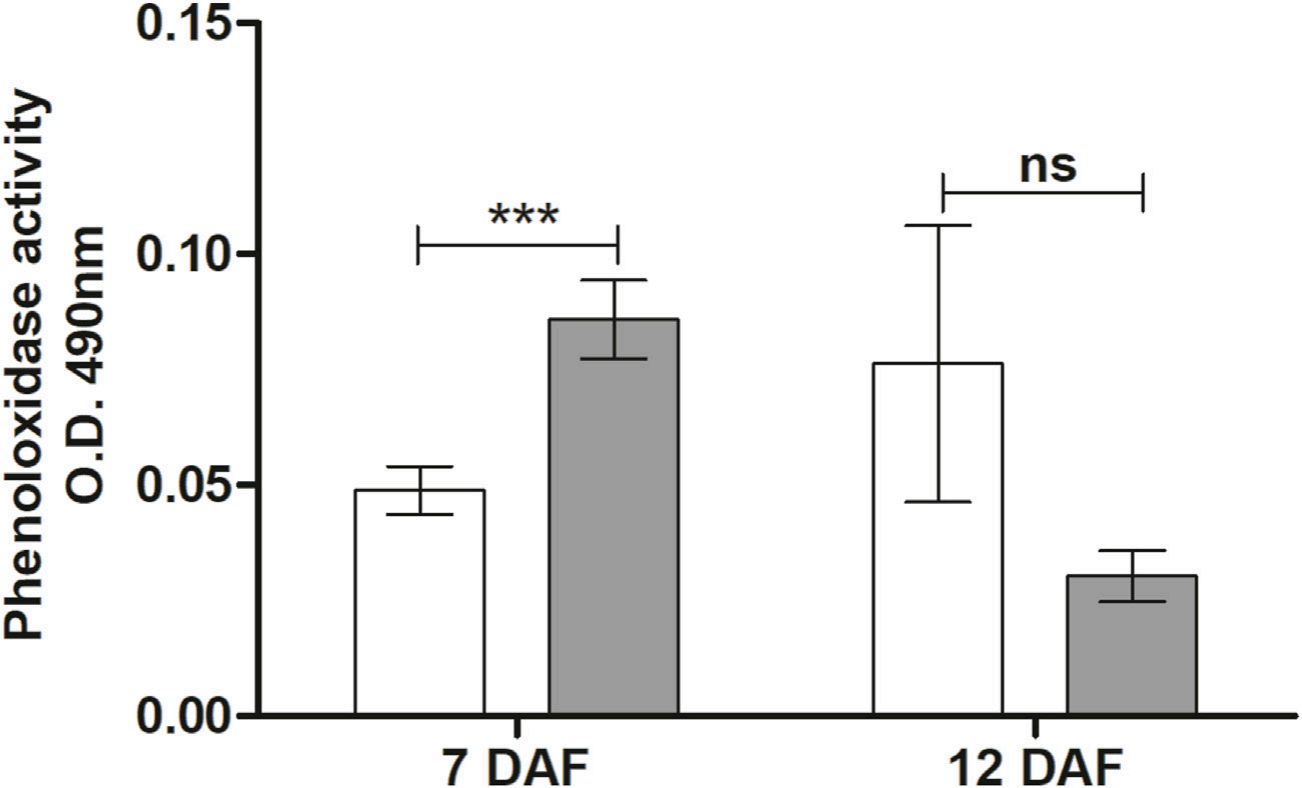
Phenoloxidase activity in the hemolymph of *Rhodnius prolixus* infected with *Trypanosoma rangeli.* Phenoloxidase activities were measured in the hemolymph collected from fifth instar nymphs of *R. prolixus* fed on blood containing *T. rangeli* epimastigotes (10^6^ parasites/mL of blood) on different days after feeding (7 and 12 DAF). White column - control, not infected insects; grey column - insects infected with *T. rangeli*. Each data represents the mean of three independent experiments with five insects in each group (n = 15) per day analyzed. The vertical bars indicate the standard error (±SEM). Groups were compared for each day, using the *t*-test, n = 15 insects. ***p < 0.001 and ns = Non-significant difference.

Phenoloxidase activity in the hemolymph *of R. prolixus* infected by *T. rangeli* was examined at 7 and 12 DAF. At 7 DAF, there was a significant increase in PO activity in *T. rangeli* infected insects (*p* < 0.001) compared to the control group and a tendency without statistical significance to decrease PO activity at 12 DAF (Figure 6).

### Changes of *R. prolixus* midgut microbiota composition after *Trypanosoma rangeli* infection

**FIGURE 7:**
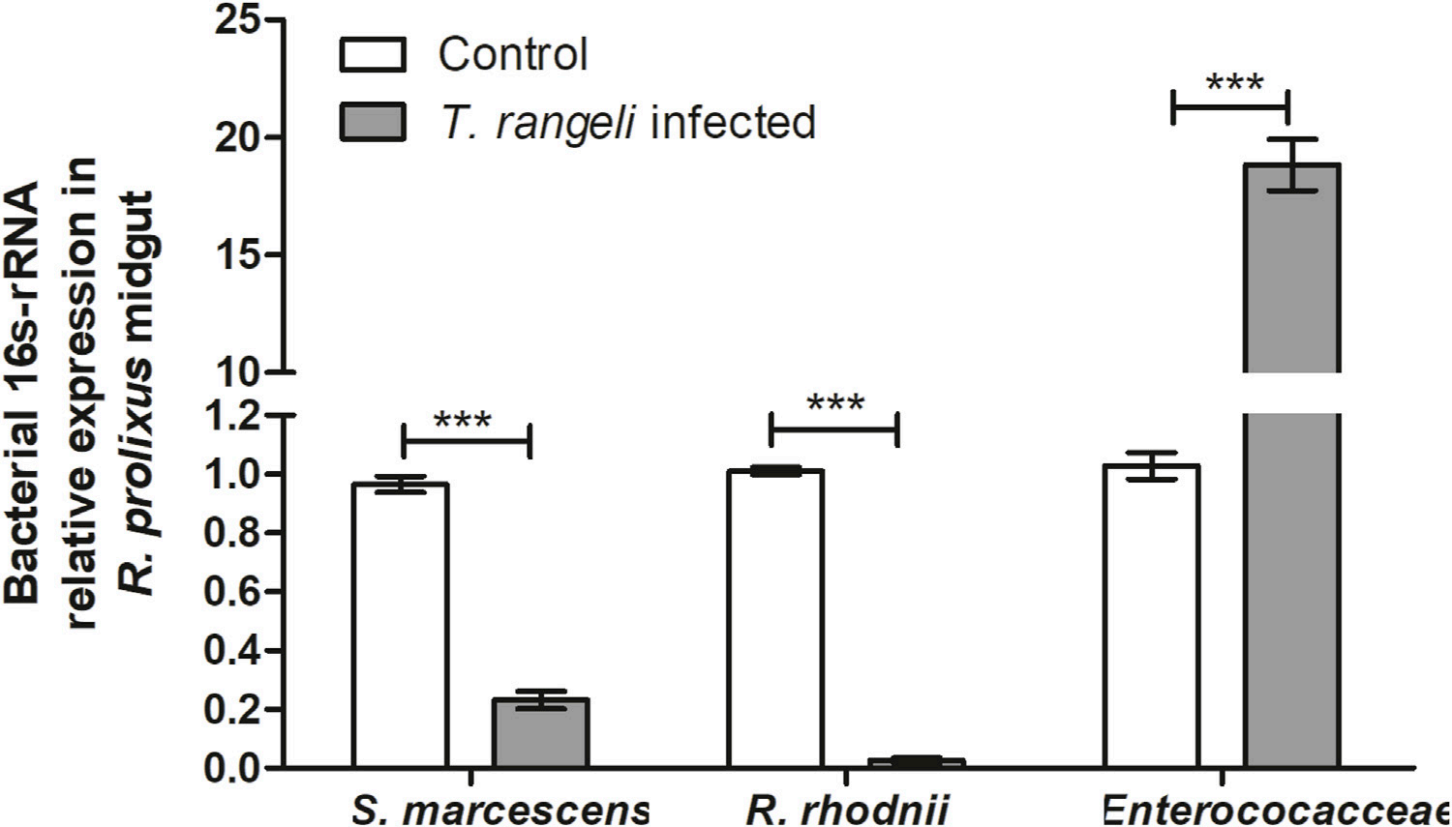
*Trypanosoma rangeli* infection modifies bacterial microbiota abundance in *Rhodnius prolixus* midgut. Determination of bacterial load in the midgut of *R. prolixus* fifth instar nymphs fed with blood containing *T. rangeli* epimastigotes (10^6^ parasites/mL). The relative expression of 16S-rRNA of *Serratia marcescens*, *Rhodococcus rhodnii*, and Enterococcaceae was *evaluated* by RT-qPCR. Data were normalized to the *R. prolixus* 18S RNA gene and calculated using the gene expression of control insects fed on blood as the calibrator. White column - control insects fed on blood; gray column–insects fed on blood containing *T. rangeli*. Bars represent the mean ± SEM of three independent experiments with ten insects (n = 30). Means were compared using the Student’s T-test; ***p < 0.001.

The *R. prolixus* bacterial gut microbiota population was quantified 7 days after *T. rangeli* infection. According to qPCR the populations of *S. marcescens* (*p* < 0.001) and *R. rhodnii* (*p* < 0.001) were significantly reduced (Figure 7). On the other hand, bacteria from the Enterococcaceae family exhibited a remarkable population increase in the midgut of insects infected with *T. rangeli* (*p* < 0.001) (Figure 7).

### The *Serratia marcescens* RPH1 trypanolytic activity against *Trypanosoma rangeli*

**FIGURE 8:**
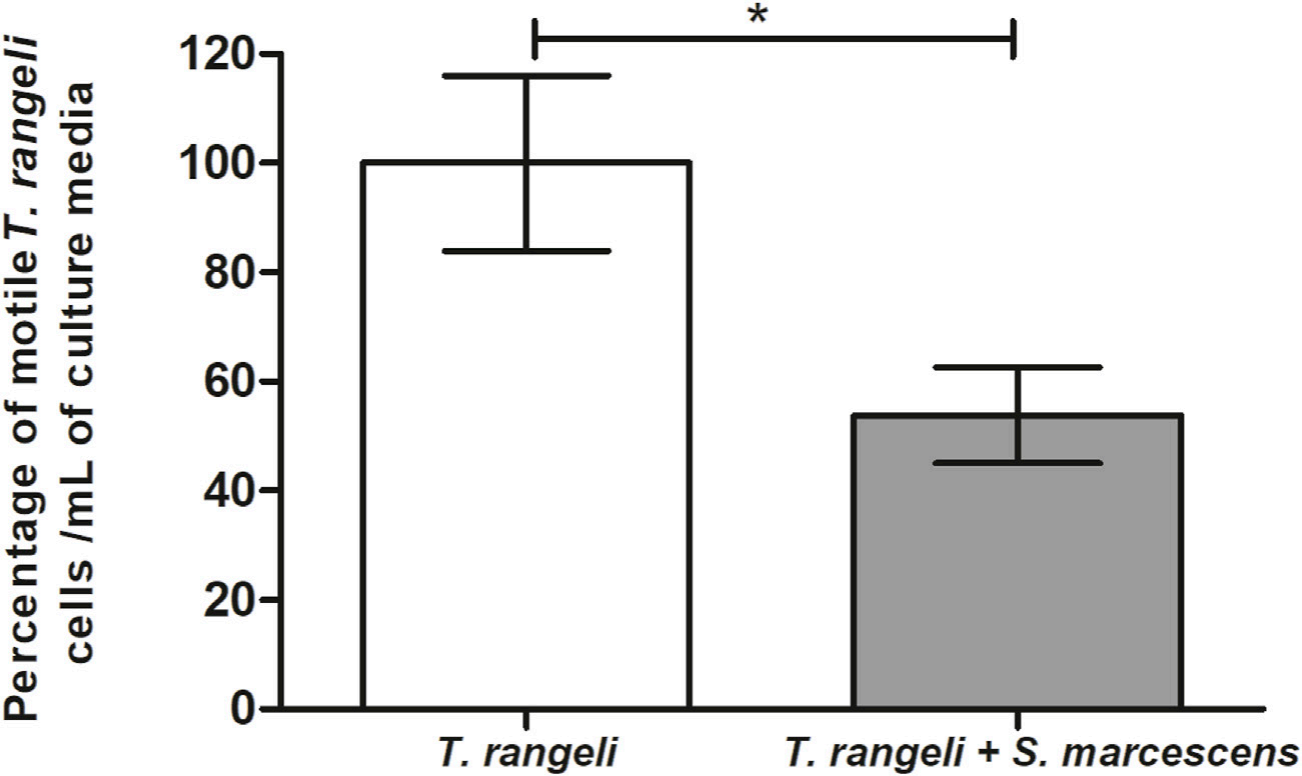
Trypanolytic activity of *Serratia marcescens* RPH1 on *Trypanosoma rangeli*. The trypanolytic activity of *S. marcescens* (S.m) RPH1 strain against *T. rangeli* (T.r) epimastigotes was tested by incubating both microorganisms for 2 hours at 30°C. The vertical axis represents the percentage of intact parasites/mL compared to the controls without the bacteria (0%). Bars represent the mean ± SEM of three independent experiments. The asterisks above the bars indicate statistical significance calculated using a *t*-test. *p < 0.05.

The lytic activity of *S. marcescens* RPH1 was tested *in vitro* against the *T. rangeli* Macias strain. Two hours after parasite incubation with *S. marcescens*, a significant trypanolytic effect against *T. rangeli* was observed, resulting in a decrease of 50% in the parasite population (*p* < 0.05) (Figure 8).

## Discussion

Detecting non-self-components is crucial to ensure animal survival. Insect vectors have the difficult task of dealing with parasite infection, immune system activation, and microbiota maintenance, an uneasy balance that could cost insect fitness and life span. Primarily, the insects rely on phylogenetically germ-line encoded recognition molecules within their innate immunity to identify and kill microbes, thereby avoiding local and systemic infections (Janeway and Medzhitov, 2002; Ferrandon et al., 2007). In nature, *R. prolixus* could be infected by *T. cruzi*, *T. rangeli,* or both simultaneously (Ramirez et al., 2002; Castillo-Castañeda et al., 2022; Herrera et al., 2022; Vergara-Meza et al., 2022). The molecular mechanisms used by *R. prolixus* to recognize *T. rangeli* infection and how the parasite modulates specific insect immune effectors and gut microbiota have not been fully explored. However, *T. cruzi* is recognized by *R. prolixus* through NF-kB signaling pathways (Vieira et al., 2018), which ultimately leads to the activation of AMP, PO cascades, as well as RNS and ROS in insect gut (Castro et al., 2012; Vieira et al., 2016; Vieira et al., 2018; Batista et al., 2020). Notably, the Toll and IMD pathways have been the most studied immune signaling pathways *in R. prolixus* (Mesquita et al., 2015; Vieira et al., 2018; Vieira et al., 2021; Salcedo-Porras et al., 2019; Salcedo-Porras and Lowenberger, 2019). The present study sheds light on the modulation of Toll and IMD signaling pathways in *R. prolixus* infected with *T. rangeli* not only in the midgut (local immune response) but also in the fat body and salivary glands (systemic and local immune responses) and the effects on AMP expression, PO activity, hemocyte microaggregation, and microbiota homeostasis.

Even though *R. prolixus* possesses a well-orchestrated mechanism for eliminating invading pathogenic microorganisms (Azambuja et al., 2017; Salcedo-Porras and Lowenberger, 2019), *T. rangeli* establishes infection in insect midgut as epimastigotes and activate *R. prolixus* local immune responses (Vieira et al., 2015; Rolandelli et al., 2021) which in turn affects insect microbiota (Vieira et al., 2015). Before invading the insect hemocoel, the *T. rangeli* oral infection leads to immunosuppression of the cellular and humoral immune responses (Whitten et al., 2001; Gomes et al., 2003; Garcia et al., 2004; Figueiredo et al., 2008). This process of hemocoel’s immune response modulation before the parasite invasion is probably correlated to interorgan communication and participation of signaling molecules such as nitric oxide and eicosanoids, as observed in mosquitos (Das et al., 2018). Then, these epimastigotes traverse the midgut epithelium, invade the insect hemolymph, and evade *R. prolixus* immune responses through various molecular mechanisms (Mello et al., 1995; Garcia et al., 2012; Peterson and Graham, 2016; Ferreira et al., 2018). Finally, the parasite infiltrates salivary glands to complete its life cycle in the invertebrate host (Ellis et al., 1980; Azambuja and Garcia, 2005; Paim et al., 2013).

Herein, we could observe a successful infection of the *T. rangeli* Macias by encountering parasites in salivary glands of *R. prolixus.* Although we found few insects with successful infection in salivary glands, several studies demonstrate that crossing the hemocoel, which has potent immune responses, is a bottleneck for developing *T. rangeli*. Hemolymph infections can occur at frequencies from 2% to 50% in orally infected insects (Hecker et al., 1990). Furthermore, the detection of *T. rangeli* in salivary glands may vary concerning the days after infection, ranging from 5 to 40 days, depending on the strain of the parasite and the insect vector species (Hecker et al., 1990; Ferreira et al., 2010; Garcia et al., 2012; Paim et al., 2013). This variation in the success of parasite development is related to the capacity of the parasite to modulate the insect’s immune responses (Castro et al., 2012; Vieira et al., 2016).

Oral infection by *T. rangeli* in *R. prolixus* modulates cellular immune responses such as microaggregation and phagocytosis by inhibiting the enzyme phospholipase A2, which initiates the eicosanoids and platelet activation factor pathways (Garcia et al., 2004; Garcia et al., 2009; Machado et al., 2006; Figueiredo et al., 2008; Kim et al., 2018). However, no studies have demonstrated the recognition of *T. rangeli* by *R. prolixus* pattern recognition receptors, in general, related to Toll or IMD pathways.

In this work, we detected the Toll pathway activation by significantly increasing *Rpdorsal* TF expression in the midgut and fat body of infected insects with *T. rangeli* Macias. Furthermore, its inhibitor (*Rpcactus*) expression was reduced on nymphs’ midgut 1 DAF. Therefore, the recognition of *T. rangeli* activates the Toll pathway by reducing the TF inhibitor, allowing more efficient *Rpdorsal* TF translocation to the nucleus. This modulation causes an increase in effector molecule synthesis, such as antimicrobial peptides, for parasite control. The Toll pathway is well conserved in *R. prolixus* (Mesquita et al., 2015); in its genome, the presence of homologous death-domain and protein adaptor genes corresponding to NF-KappaB has been observed (Zumaya-Estrada et al., 2018; Nishide et al., 2019; Salcedo-Porras and Lowenberger, 2019).

When *R. prolixus* was orally treated with IMD-0354, a drug that blocks NF- KappaB translocation to the nucleus and challenged with *E. coli* or *Serratia aureus,* it was a rapid increase in *Rp-Cactus* expression (1 DAF) (Vieira et al., 2018). Lately, 7 days after bacterial challenge, the mRNA levels of *Rpcactus* decreased significantly in the vector’s midgut (Vieira et al., 2018). In this context, we note an autoregulation profile on inhibitor expression, where the parasite is initially present. *T. rangeli* triggers an effective expression of *Rpcactus* with joint action of the *Rpdorsal* TF. But, during the interaction with the pathogen, the gene transcript level is controlled. In *D. melanogaster*, the Cactus gene and its isoforms are induced in the face of pathogens, being rapidly degraded when released into the cytosol but reinduced when necessary, reinforcing the autoregulation scenario in invertebrates after exposure and humoral immunity action against invading pathogen (Nicolas et al., 1998).

When observing a reduction of *Rp-cactus* in the midgut, it is essential to regard that this is an important region for *T. rangeli* development since it is where the parasite migrates from midgut to hemolymph and that the Toll pathway activation triggers a cascade of signals inducing effector genes transcription and pathogen control (Zumaya-Estrada et al., 2018).

The parasite must reach the insect’s salivary glands to effectively complete the *T. rangeli* life cycle and subsequent transmission to a vertebrate host. Notably, activating immune responses in the midgut directly influences the parasite’s ability to establish a successful infection. Prolonged infections with *T. rangeli* strain CHOACHI revealed a notable observation: insects hosting the parasite in their gut, rather than in the hemolymph, exhibited a significant upregulation of *Rpcactus* inhibitor transcription in the midgut (Rolandelli et al., 2021). Therefore, diverse *T. rangeli* strains may distinctly impact specific aspects of humoral immunity in the vector. In the present work, we observed IMD pathway activation, an *R. prolixus* pathway not wholly identified in the genome (Mesquita et al., 2015). However, bioinformatic analyses later identified these elements in *R. prolixus* (Salcedo-Porras and Lowenberger, 2019) and other triatomine species (Zumaya-Estrada et al., 2018). Intent to explore a primary immune response by evaluating *T. rangeli* Macias infection, *Rprelish* TF expression was not modulated on both fat body and midgut. However, knowing that pathogen recognition patterns mediate the initial recognition of an infection, we also evaluate *PGRP* expression, which is a receptor associated with IMD pathway activation. Since *RpPGRP* expression in both midgut and fat body 1 DAF was increased, these results indicate that *T. rangeli* infection in *R. prolixus* is recognized by the expression of PGRPs. A reduction in AMP expression was shown after PGRPs silencing the Hemiptera *Plautia stali* and its infection by Gram + and Gram–bacteria (Nishide et al., 2019). *R. prolixus Rprelish* TF silencing controls humoral effectors, leading to Defensin C inhibition in insects challenged with Gram + and Gram - bacteria (Salcedo-Porras and Lowenberger, 2019), as well as reduced defensin A and increased lysozyme A in the midgut of insects challenged with *T. cruzi* (Mesquita et al., 2015).

The expression of AMPs directly depends on the activation of signaling pathways. *R. prolixus* challenged with *T. rangeli* Macias presents an increase in the expression of *RpdefC* in fat body 1 and 7 DAF, while in salivary glands the upregulation occurred at 7 DAF. When *R. prolixus* was challenged with *T. cruzi* Dm28c, the expression of these AMPs was also shown to be elevated in the midgut of insects 7 DAF (Vieira et al., 2018). In contrast, when these insects are challenged by Gram + and Gram - bacteria, *RpdefC* expression is increased in the midgut at 24 h post infection but reduces over days (Vieira et al., 2014). This dynamic pattern of AMP expression reflects the insect’s response to different types of pathogens over time.

Still, little is known about which signaling pathway is responsible for expressing such specific AMPs in *R. prolixus* to control the invading organism. In our results, *T. rangeli* stimulates *Rp-dorsal* expression and inhibits *Rpcactus*. Consequently, we observed a significant increase in *RpdefC* expression. Oppositely, the parasite negatively modulated *RpdefA*, *RpdefB*, and *Rpprol*. Together, these results indicate that *T. rangeli* activates the Toll pathway in *R. prolixus*, which regulates the expression of *RpdefC*, in agreement with the previous publication (Vieira et al., 2015). On the other hand, *T. rangeli* infection seems to inhibit the IMD pathway, which explains the suppression of *RpdefA* and *RpdefB*.

Regarding cellular immune responses modulated by *T. rangeli* Macias oral infection in *R. prolixus*, we observed increased PO activity and nodule formation. The prophenoloxidase system is a rapid response process that occurs independently of gene expression (Cerenius et al., 2010). It uses the enzyme PO to produce enzymatic groups polymerized into melanin. These events are triggered when an insect suffers an injury or infection by a pathogen, leading to the melanization of invading microorganisms (Söderhäll and Cerenius, 1998; Christensen et al., 2005; González-Santoyo and Córdoba-Aguilar, 2012). Triatomines respond differently depending on the challenge they receive. The presence of *T. rangeli* in *R. prolixus* hemolymph inhibits PO activity (Gregório and Ratcliffe, 1991). When *R. prolixus* was inoculated with short epimastigotes of *T. rangeli* strain H14, it caused an increase in PO activity. However, when oral feeding was carried out with different forms of *T. rangeli* H14 epimastigotes, it caused a suppression of the PO system (Gomes et al., 2003). *T. rangeli* Macias oral infection also reduced the PO activity in *R. prolixus*, referring to spontaneous and total PO. However, this effect is not immediately observed, occurring only 12 days after feeding (Mattos, 2014; Vieira et al., 2015). Herein, we observed increased PO activity in the hemolymph of *R. prolixus* infected with *T. rangeli* at 7 DAF and no differences at 12 DAF compared to non-infected insects. Although we did not detect a significant difference, 12 DAF, an apparent reduction in PO activity can be observed.

Also, in the hemocele, the hemocytes can aggregate, forming nodules to fight microorganisms (Ratcliffe and Gagen, 1977; Satyavathi et al., 2014). We investigated hemocyte recruitment at 2 and 7 DAF. Here we observed a decrease in total free hemocytes in insects infected with *T. rangeli* at 12 DAF, compared to control insects. The formation of nodules depends on the aggregation of hemocytes, which reduces the presence of circulating hemocytes in the hemolymph. At 7 and 12 DAF, insects infected with *T. rangeli* strain Macias showed a significant increase in the population of nodules present in the hemolymph of *R. prolixus,* which could explain the decrease in hemocytes number at 12 DAF in infected insects compared to control.

*In vitro*, infection by *T. rangeli* stimulates the formation of nodules in *R. prolixus* hemolymph. However, when the *T. rangeli* infection occurs *in vivo*, through parasite injection into the *R. prolixus* hemocoel, there is an increase in the hemocyte population, but without significant stimulation in the formation of nodules (Gomes et al., 1999). Here, *T. rangeli* infection was performed orally through blood offered to *R. prolixus*. The presence of the parasite at 12 DAF caused a reduction in the hemocyte population and significantly increased the formation of hemocyte microaggregates (nodule formation) in *R. prolixus* hemolymph. The isolation of a lectin present in the hemolymph of *R. prolixus* impacts the motility of *T. rangeli* strain H14 and increases the formation of *T. rangeli* clusters in the hemolymph (Mello et al., 1999).

Alterations in insect immune responses can significantly impact the intestinal microbiota, particularly the modulation of antimicrobial peptides (AMPs). Our study reveals notable changes in the bacterial microbiota composition of *R. prolixus* infected with *T. rangeli* Macias, substantially reducing the populations of *R. rhodnii* and *S. marcescens*. Our results agree with former publications where the authors observed *T. rangeli* infections impact the *R. rhodnii* population in *R. prolixus* anterior midgut (Watkins, 1971; Eichler and Schaub, 2002). Previous investigations employing pyrosequencing on *R. prolixus* infected with the *T. rangeli* Macias strain did not detect differences in the number of sequences from the Nocardiaceae and Enterobacteriaceae families, associated with *R. rhodnii* and *S. marcescens* (Vieira et al., 2015). However, a decrease in Enterococcaceae and an increase in Burkholderiaceae were documented in *T. rangeli* infected insects (Vieira et al., 2015). Interestingly, a reduction in *S. marcescens* and *R. rhodnii* populations was observed in the midgut of *R. prolixus* infected with the *T. cruzi* Dm28c strain (Vieira et al., 2016).

Once AMP expression is differentially regulated in the midgut depending on the species and strains of parasite-infected insects, it impacts the microbiota composition inside the intestinal tract, where certain species are negatively modulated, and others are positively modulated.

Defensin C is correlated with bacterial microbiota regulation of *R. prolixus* (Vieira et al., 2015; Vieira et al., 2016; Vieira et al., 2018; Vieira et al., 2021). Vieira et al. (2015) observed an increase in *RpdefB* and *RpdefC* levels and a decrease of cultivable bacteria in the anterior midgut in short-term *T. rangeli* Macias strain infected *R. prolixus*. Moreover, in *T. rangeli*, a long-term infection caused a massive upregulation of *RpdefC* in the posterior midgut and decreased the bacteria population (Vieira et al., 2015). In *R. prolixus* infected with *T. cruzi* Dm28c, there was an increase in *RpdefC* and *Rpprol* expression levels in the anterior midgut and a drastic reduction of *S. marcescens* and *R. rhodnii* 16S gene expression (Vieira et al., 2016). Immunodepression of *R. prolixus* by treating the insects with IMD-0354, a selective inhibitor of IkB kinases, downregulated the expression of defensins, caused a reduced antibacterial activity of the insect anterior midgut against *S. marcescens* and an intense proliferation of the bacteria detected by 16S-RNA relative expression of *S. marcescens*, *R. rhodnii* and bacteria of the Enterococacceae family (Vieira et al., 2018). Also, the immune depression of *R. prolixus* by treatment with azadirachtin, an ecdysone inhibitor, caused a reduction in the transcription level of *RpdefC* (almost 200-fold) and an increase in the load of *S. marcescens* 16SRNA expression level (Vieira et al., 2021).

Considering all these observations collectively, it can be inferred that *T. rangeli* infection triggers the activation of the Toll signaling pathway in *R. prolixus*, thereby inducing the synthesis of defensin C, which plays a pivotal role in modulating the intestinal microbiota, particularly targeting *S. marcescens*. The relationship between gut-microbiota and pathogens transmitted by insect vectors is complex and it can be observed in different types of vectors, where pathogens can modify the microbial load in the midgut and/or the composition of the bacterial population (Gabrieli et al., 2021).

This complex modulation of the insect’s immune system and microbiota through *T. rangeli* infection could be the reason for the impact observed on the insect’s physiology as a trade-off (Guarneri et al., 2017; Ferreira et al., 2018).

## Conclusion

In the present investigation, it became clear that the activation of the vector humoral immunity plays a pivotal role in the success of *T. rangeli* infection. *T. rangeli* Macias seems to be recognized mainly by the Toll pathway, which regulates *RpdefC* expression. Immunity modulation by *T. rangeli* substantially influences the population dynamics of *S. marcescens* in the insect midgut, thereby helping parasite evasion of the toxic trypanolytic effects exerted by microbiota, favoring parasite establishment in the vector. Parallel, during its invasion into the insect hemocele, *T. rangeli* distinctly engages with *R. prolixus* cellular immunity. Notably, *T. rangeli* leads to a marked reduction in the population of circulating hemocytes, accompanied by a significant increase in nodule formation. Also, even inside insect salivary glands, *T. rangeli* faces the activation of AMPs. Despite the activation of insect immune responses and release of immune effectors, *T. rangeli* successfully crosses the triatomine’s midgut, colonizes the insect’s hemolymph, and invades salivary glands, completing its life cycle.

## Data Availability

The original contributions presented in the study are included in the article/Supplementary Material, further inquiries can be directed to the corresponding author.
