# Supplementary material for: Immune signaling pathways in Rhodnius prolixus in the context of Trypanosoma rangeli infection: cellular and humoral immune responses and microbiota modulation
Source: Front Physiol. 2024 Aug 15;15:1435447. doi: 10.3389/fphys.2024.1435447 (PMC11357937; doi:10.3389/fphys.2024.1435447)
Supplement: Supplementary file 1 [file Table1.docx]

**Supplementary Table 1: Oligonucleotide primers used for RT-qPCR analysis**

| **Gene/Name** | **Sequence 5’-3’** | **Amplicon length** | **Reference** |
| --- | --- | --- | --- |
| GAPDH-F  GAPDH-R | GATGGCGCCCAGTACATAGT  AGCTGACGGGGCTGTTATTA | 111pb | Paim et al., 2012 |
| TUB-F  TUB-R | TTTCCTCGATCACTGCTTCC  CGGAAATAACTGGGGCATAA | 129pb | Paim et al., 2012 |
| RPDEFA- F  RPDEFA-R | GAATACTCCACTCAACCGCAAC  AGGGCATCATCTAGTTGTTGATGAGTG | 131pb | Vieira et al., 2016 |
| RPDEFB-F  RPDEFB-R | GGATATTCCACTCAACCGCAAC  AGAGCATCGTCTAATTCTTGTTGAGTG | 131pb | Vieira et al., 2016 |
| RPDEFC-F  RPDEFC-R | CAGTACAGTCCTAATACCTAGCC  TGGGCATCATCTAATTGATGTTGAGAA | 136pb | Vieira et al., 2016 |
| PROL-F  PROL-R | CTATAACGAGTGAACTATAAGACAA  GTGTTTAATGGCGGTAACAAATTAC | 406pb | \| Ursic-Bedoya et al., 2011 \| \| --- \| |
| RpDorsal-F  RpDorsal-R | CAACAGCTGCTAAACCGACA  CAATGGTCGTTCTTGGACT | 77pb | Mesquita et al., 2015 |
| RpRelish-F  RpRelish-R | TTTTTCGTGAGCAACTGGTG  GCAGCCCCAAAGTTCTTACA | 76pb | Mesquita et al., 2015 |
| RpCactus- F  RpCactus-R | \| GTGCTGGTGCTTGTACGAAA \| \| --- \|   GGAGTCGGACGATACCTCAA | 78pb | Mesquita et al., 2015 |
| 16S *S. marcescens* - F  16S *S. marcescens* - R | GGTGAGCTTAATACGTTCATCAATTG  GCAGTTCCCAGGTTGAGCC | 179pb | Saikaly et al., 2007 |
| 16S *R. rhodnii*-F  16S *R. rhodni*i-R | CACTGGTTGCATGGCCTGGTG  TGAGCTGTGGGATTTCACAGAC | 418pb | Vieira et al., 2016 |
| Enterococcaceae -F Enterococcaceae - R | CCCATCAGAAGGGGATAACACTT  ACCGCGGGTCCATCCATC | 426pb | Vieira et al., 2018 |
