# Supplementary figures and images for: Immune signaling pathways in Rhodnius prolixus in the context of Trypanosoma rangeli infection: cellular and humoral immune responses and microbiota modulation
Source: Front Physiol. 2024 Aug 15;15:1435447. doi: 10.3389/fphys.2024.1435447 (PMC11357937; doi:10.3389/fphys.2024.1435447)

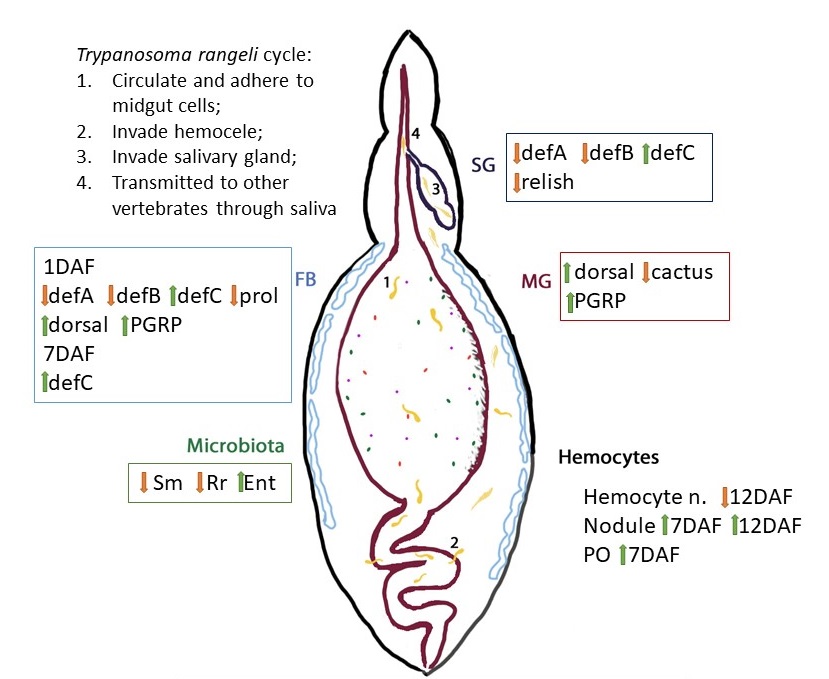

Supplement: Supplementary file 2 [file Image1.JPEG]
